# Synthesis of oligonucleotides on a soluble support

**DOI:** 10.3762/bjoc.13.134

**Published:** 2017-07-12

**Authors:** Harri Lönnberg

**Affiliations:** 1 Department of Chemistry, University of Turku, FIN-20014 Turku, Finland

**Keywords:** DNA, oligonucleotides, RNA, soluble support, synthesis

## Abstract

Oligonucleotides are usually prepared in lab scale on a solid support with the aid of a fully automated synthesizer. Scaling up of the equipment has allowed industrial synthesis up to kilogram scale. In spite of this, solution-phase synthesis has received continuous interest, on one hand as a technique that could enable synthesis of even larger amounts and, on the other hand, as a gram scale laboratory synthesis without any special equipment. The synthesis on a soluble support has been regarded as an approach that could combine the advantageous features of both the solution and solid-phase syntheses. The critical step of this approach is the separation of the support-anchored oligonucleotide chain from the monomeric building block and other small molecular reagents and byproducts after each coupling, oxidation and deprotection step. The techniques applied so far include precipitation, extraction, chromatography and nanofiltration. As regards coupling, all conventional chemistries, viz. phosphoramidite, *H*-phosphonate and phosphotriester strategies, have been attempted. While P(III)-based phosphoramidite and *H*-phosphonate chemistries are almost exclusively used on a solid support, the “outdated” P(V)-based phosphotriester chemistry still offers one major advantage for the synthesis on a soluble support; the omission of the oxidation step simplifies the coupling cycle. Several of protocols developed for the soluble-supported synthesis allow the preparation of both DNA and RNA oligomers of limited length in gram scale without any special equipment, being evidently of interest for research groups that need oligonucleotides in large amounts for research purposes. However, none of them has really tested at such a scale that the feasibility of their industrial use could be critically judged.

## Introduction

**Figure 1:**
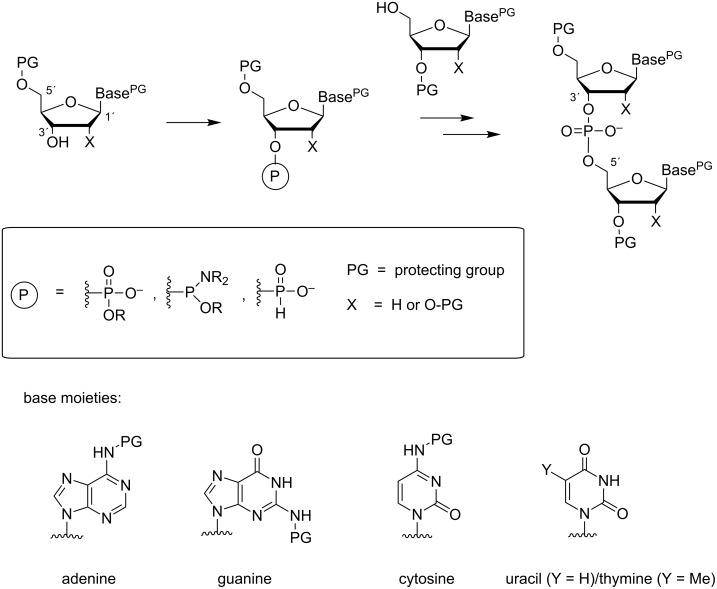
General principle of oligonucleotide synthesis.

**Scheme 1:**
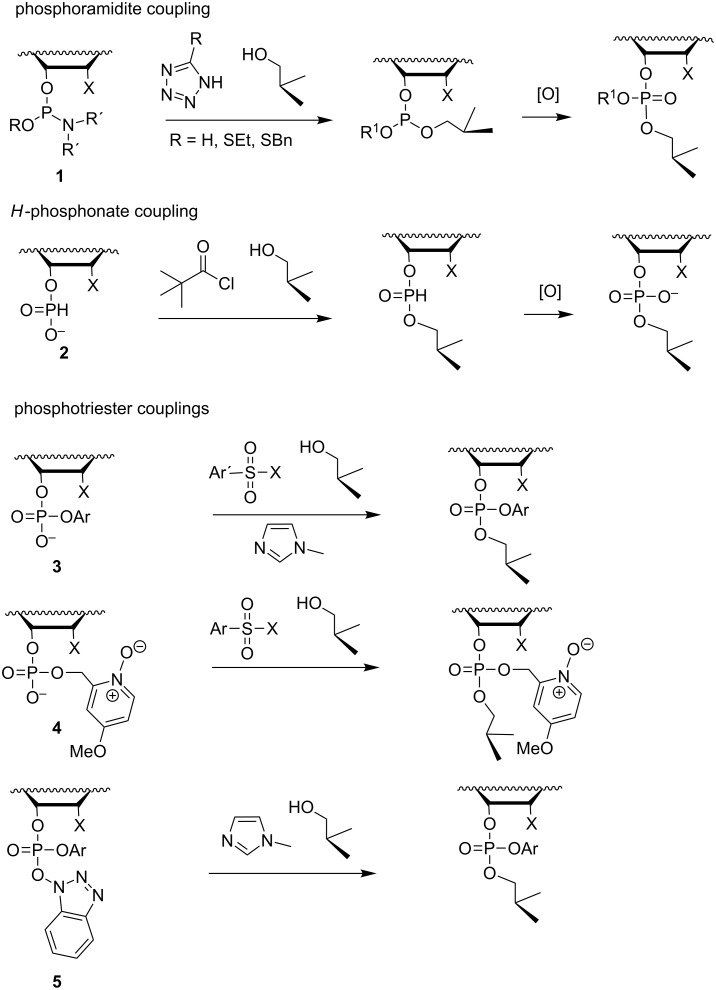
Alternative coupling methods used in the synthesis of oligonucleotides.

The synthesis of oligonucleotides (ONs) consists of linking nucleosides to each other in a specified order by esterification of phosphoric acid with the 3´-OH of one and the 5´-OH of the other nucleoside. Usually, the 3´-OH is first esterified with an appropriate derivative of phosphoric acid and the resulting building block is then reacted with the 5´-OH (Figure 1). Either a linear or a convergent strategy may be utilized, but the stepwise linear approach proceeding from the 3´- to the 5´-terminus of ON is nowadays almost exclusively exploited [1–2]. The coupling reaction may take place either at oxidation level III or V of phosphorus. Owing to higher reactivity of P(III) centers, appropriately protected nucleoside 3´-(2-cyanoethyl-*N*,*N*-dialkylphosphoramidite)s (**1** in Scheme 1) or 3´-(*H*-phosphonate)s are usually preferred as building blocks [3] (**2** in Scheme 1). The attacking 5´-OH apart, all other nucleophilic functionalities must be kept protected during the coupling. The primary amino groups of the nucleobases are usually protected with acyl groups and the 5´-OH of the monomeric building block with a 4,4’-dimethoxytrityl group (DMTr), or sometimes with its monomethoxytrityl analog (MMTr) [4–5].

To achieve coupling, phosphoramidites are activated with azoles [6], such as tetrazole [7], its derivatives 2-ethyl- and 2-benzylthiotetrazole [8] or 4,5-dicyanoimidazole [9]. The activator has a dual role donating a proton to the departing dialkylamino group and attacking as an anionic species on phosphorus [10]. Nucleoside *H*-phosphonates are, in turn, converted in situ to reactive mixed anhydrides with acyl chlorides or chlorophosphates [11–13]. On applying the phosphoramidite chemistry, the phosphite triesters obtained are oxidized to phosphate triesters in each coupling cycle, whereas the *H*-phosphonate diesters may be stable enough to become oxidized only at the end of chain assembly. When the coupling is carried out at P(V) level, 3´-arylphosphate diesters (**3** in Scheme 1) are normally used as building blocks and activated with arylsulfonyl chloride or azolide in the presence of an auxiliary nucleophilic catalyst [14], or a catalytically active phosphate protecting group, such as the 4-methoxy-1-oxido-2-picolyl group [15], is used instead of a non-participating arylphosphate group (**4** in Scheme 1). Alternatively, prefabricated or in situ activated 1-hydroxybenzotriazole 3´-arylphosphotriesters may be used for coupling in the presence of a nucleophilic catalyst [16–17] (**5** in Scheme 1).

Compared to oligodeoxyribonucleotides (ODNs), the synthesis of oligoribonucleotides (ORNs) is complicated by the presence of an additional nucleophilic functionality, viz. the 2´-OH that has to be kept protected as long as basic conditions are required during synthesis and deprotection of the oligonucleotide. Since the phosphate protecting groups are normally base-labile and the repeatedly removable 5´-*O* protecting group is acid-labile, the 2´-*O*-protection should preferably be removable under orthogonal conditions. For this purpose, numerous protecting groups have been proposed [18–19], the fluoride ion labile *tert*-butyldimethylsilyl (TBDMS) [20]) and triisopropylsilyloxymetyl (TOM) [21] groups being most widely used. Otherwise, the synthetic strategies are similar to those of ODNs.

The real breakthrough of the chemical synthesis of oligonucleotides was the finding of Beaucage and Caruthers in the early 1980s, according to which appropriately protected nucleosides could rapidly be coupled as 3´-(*O*-alkyl-*N*,*N*-dialkylphosphoramidite)s to 5´-OH of a support bound nucleoside by using tetrazole as an activator [7]. Since then, this solid-supported phosphoramidite chemistry has almost exclusively used for the preparation of oligonucleotides from lab scale [3,22] to industrial synthesis up to kilogram scale [23]. In spite of the obvious success of this methodology, synthesis in solution phase has received continuous interest as an alternative for large-scale synthesis, and the recent advances in the development of therapeutic oligonucleotides targeting either pre-mRNA [24–25], mature mRNA [26–28] or noncoding microRNA [29–30] have even increased this interest. It has been repeatedly argued that (i) the synthesis in solution could be carried out with a smaller excess of building blocks, (ii) the scale up procedure would be more straightforward and (iii) expensive solid support material is not needed. In addition, the possibility to characterize the growing chain by mass or NMR spectroscopy after each coupling is an attractive feature, although not possible with all soluble supports. While major advances in the large scale solid-phase technology have been taken, the difference in the consumption of building blocks in solution and on a solid-support is not necessarily as substantial as previously assumed; the phosphoramidite-chemistry-based synthesis has been optimized to the level that building blocks are required only in a moderate excess, 1.5–2.0 equiv [23]. The obvious challenge is the separation of the support-anchored ON chain from small molecular reagents after each coupling cycle, a step that on a solid-support can be carried out by simple washing. Precipitation, chromatography, extraction and nanofiltration have been considered to be feasible approaches.

Even if the synthesis on a soluble support fails to compete with industrial solid-phase synthesis, it may still play an important role in up to gram scale laboratory synthesis, since no special equipment is usually needed. Spectroscopic studies on structure, dynamics and recognition of ONs by other biopolymers, small molecules or metal complexes, for example, may consume ONs in amounts that cannot be conveniently reached by lab-scale solid-phase synthesizers. In addition to synthesis on a soluble support, impressive examples of classical convergent synthesis [31–34] and exploitation of solid-supported reagents in solution [35–36] have been reported. The present review, however, surveys only the progress of ON synthesis on a soluble support.

## Review

### Synthesis of oligodeoxyribonucleotides by phosphotriester chemistry

**Scheme 2:**
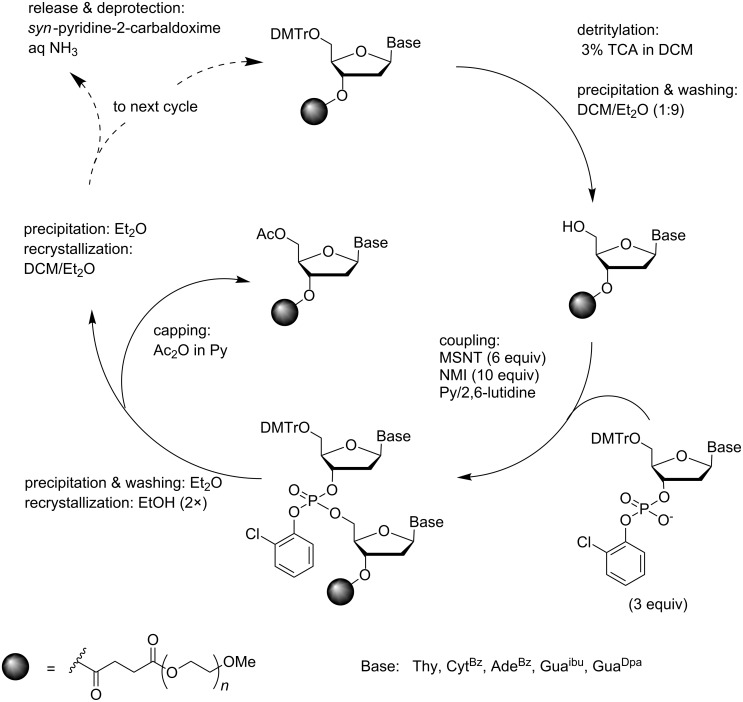
Synthesis of ODNs on a precipitative PEG-support by phosphotriester chemistry using MSNT/NMI activation [37].

The pioneering syntheses of ONs on a soluble support were carried out by the phosphotriester strategy. Although this coupling chemistry is seldom used on a solid support where small molecule reagents and wastes can be removed by simple washing, the avoidance of the oxidation step due to use of P(V) synthons markedly simplifies the coupling cycle. This is a marked advantage in case of solution synthesis where the excess of reagents and wastes must be removed by a more laborious technique. The first synthesis of a reasonably long ODN, viz. an octamer d(5´-TAGCGCTA-3´), was carried out by Bonora et al. [37] on polyethylene glycol (PEG 5000) monomethyl ester. The overall strategy was rather similar to that of the solid-supported chemistry (Scheme 2). Accordingly, the 3´-terminal nucleoside, 5´-*O*-DMTr-*N*^6^-Bz-dA, was attached to the support via a 3´-succinyl linker, the 5´-*O*-DMTr group was removed with 3% TCA in DCM and the derivatized support was isolated by precipitation with Et_2_O and recrystallization from a 1:9 (v/v) mixture of DCM and Et_2_O. 5´-*O*-DMTr-nucleosides (3.0 equiv of dT, dC^Bz^, dG^ibu^, dG^Dpa^, dA^Bz^) were then coupled as 3´-(2-chlorophenylphosphate)s in a mixture of pyridine and 2,6-lutidine using 1-(mesitylene-2-sulfonyl)-3-nitro-1,2,4-triazole (MSNT; 6 equiv) as an activator and *N*-methylimidazole (NMI; 10 equiv) as a nucleophilic catalyst. Each coupling was followed by precipitation/recrystallization from EtOH, capping with Ac_2_O in pyridine and precipitation from DCM/Et_2_O. In spite of several precipitations and recrystallizations, one coupling cycle could be completed in 5 hours, the stepwise coupling yield ranging from 90% to 95% and the crude PEG-bound octamer was obtained in 79% yield. The coupling of dG^ibu^ proceeded, however, in more than 100% yield, which was interpreted as an indication of a side product formation. Evidently, the MSNT activation had resulted in displacement of O6 by the 3-nitro-1,2,4-triazol-1-yl group [38]. The oligomer was released from the support and deprotected by successive treatments with *syn*-pyridine-2-carbaldoxime and tetramethylguanidine in aq dioxane [39] and aq ammonia, and purified by ion-exchange chromatography on DEAE cellulose. From 980 mg of crude PEG-octamer, 85 mg of pure lyophilized TEA salt of d(5´-TAGCGCTA-3´) was obtained. In other words, the yield of the isolation step was less than 30%.

To avoid the modification of dG^ibu^ during the MSNT treatment, activation by 1-hydroxybenzotriazole, as originally introduced by Marugg et al. [40], was then attempted on the same PEG-support [41]. Accordingly, 3´-(2-chlorophenyl benzotriazol-1-yl phosphate)s of conventionally protected 2´-deoxynucleosides (3 equiv) were used as building blocks, and the coupling was carried out in a mixture of pyridine and dioxane in the presence of NMI (5 equiv). Otherwise, the protocol was similar to the previous one. The average stepwise coupling yield upon the assembly of octamer d(5´-TAGCGCTA-3´) was 93.5%, and 55% of the PEG-anchored oligomer could be isolated in pure deprotected form. No base modification reactions were now detected.

**Scheme 3:**
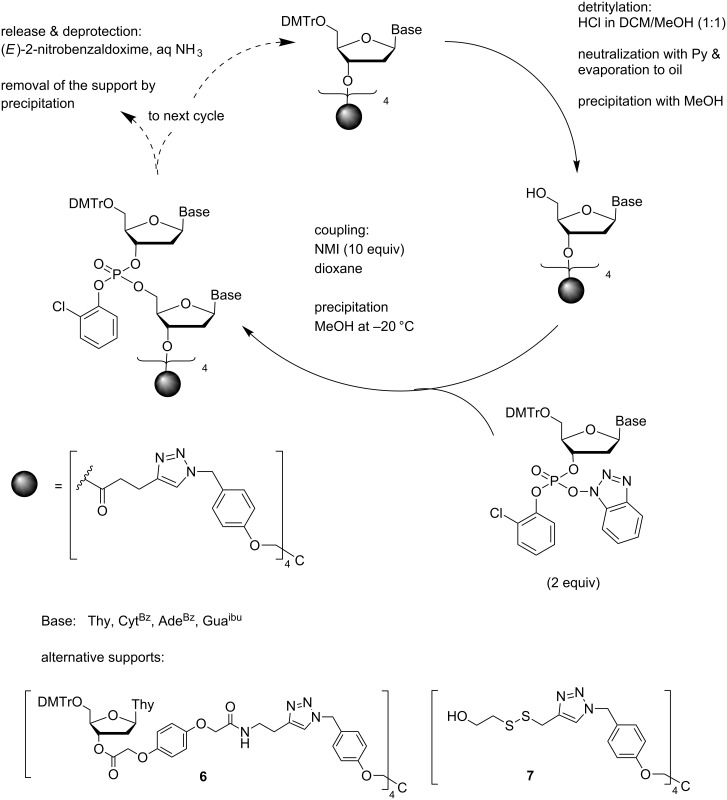
Synthesis of ODNs on a precipitative tetrapodal support by phosphotriester chemistry using 1-hydroxybenzotriazole activation [42].

The phosphotriester approach based on hydroxybenzotriazole activation has more recently applied to the synthesis of short ODNs on a branched tetrakis-*O*-[4-(azidomethyl)phenyl]pentaerythritol-derived support (Scheme 3) [42]. Owing to the symmetrical structure of the support, NMR and mass spectroscopic characterization is possible at any stage of the chain assembly. The 3´-terminal nucleoside was immobilized to this support as a 3´-*O*-(4-pentynoyl) derivative by Cu(I)-catalyzed 1,3-dipolar cycloaddition [43]. This support is soluble in MeCN and dioxane but precipitates quantitatively in MeOH. Each coupling cycle contained two precipitations, one after removal of the 5´-*O*-DMTr group and the second after the coupling step. Detritylation was catalyzed with HCl in a 1:1 (v/v) mixture of MeOH and DCM and coupling was carried out in dioxane in the presence of NMI. Precipitations were achieved by 10-fold dilution with MeOH. All small-molecule compounds remained in solution. Removal of the 2-chlorophenyl protections with the tetramethylguanidium salt of (*E*)-2-nitrobenzaldoxime in aqueous dioxane, followed by ammonolysis, removal of the support by precipitation and conversion to the sodium salt, completed the synthesis. A pentamer, d(5´-CGCAT-3´), homogeneous by HPLC, was obtained in 55% yield on using 2 equiv of building block in each coupling step. The advantages of such a tetrapodal support appear to be good atomic economy, i.e., small amount of support material compared to the amount of ORN obtained and the moderate consumption of solvent (MeOH) required for really quantitative precipitation of the support-bound oligonucleotides. However, only short oligomers have been so far prepared on this support. Support loaded with longer fully protected oligomers may precipitate less quantitatively or interchain aggregation may reduce the coupling efficiency.

A closely related support **6**, incorporating additionally a Q-linker moiety [44], has been used for preparation of fully protected ODN trimers having only the 3´-terminal hydroxy function unprotected and, hence, available for one step conversion to a phosphoramidite building block [45]. Such phosphoramidites are widely used for the assembly of ODNs useful in protein engineering by oligonucleotide directed mutagenesis [46–49]. Cleavage of the linker by 5 mmol L^−1^ K_2_CO_3_ in a 3:43:10 mixture of DCM, dioxane and MeOH (30 min), followed by neutralization with pyridinium chloride, left the 5´-*O*-DMTr group, 2-chlorophenyl phosphate protections and base moiety protections untouched. Silica gel chromatographic purification and conventional phosphitylation with 1-chloro-1-(2-cyanoethoxy)-*N*,*N*-diisopropylphosphoramidite gave the desired building blocks, the applicability of which in a solid-phase synthesis was demonstrated [45]. 3´-(2-Chlorophenyl)phosphates of protected trimeric ODNs, useful for phosphotriester coupling, have been prepared on a related reductively cleavable disulfide-linked support **7** [50].

### Synthesis of oligodeoxyribonucleotides by phosphoramidite chemistry

**Scheme 4:**
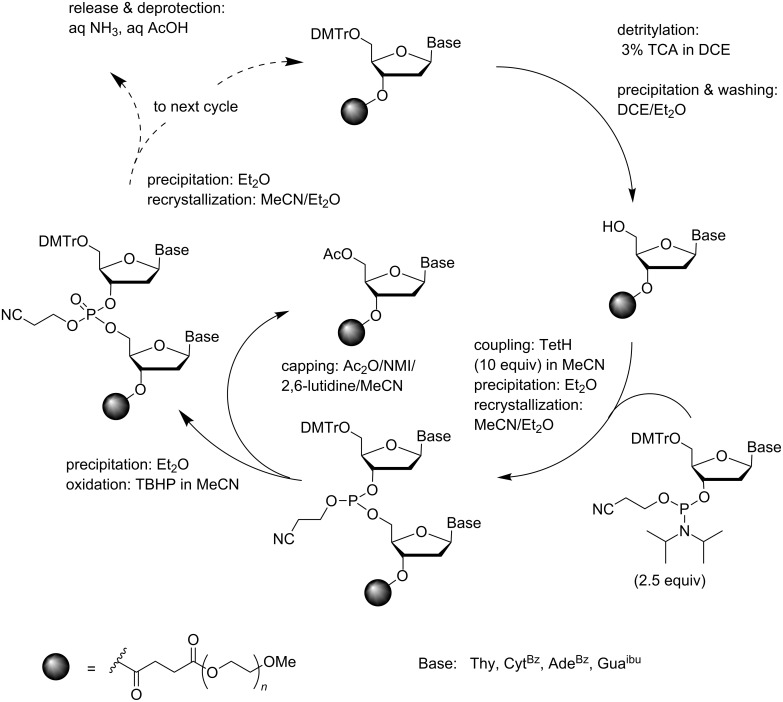
Synthesis of ODNs on a precipitative PEG-support by conventional phosphoramidite chemistry [51].

As mentioned above, phosphoramidite chemistry is nowadays the method of choice for the solid-supported synthesis of oligonucleotides both in small and large scale. The first attempt to apply the phosphoramidite chemistry to synthesis on a soluble support dates back to 1993. Both the support (PEG) and overall strategy of chain assembly were in this pioneering study of Bonora et al. [51] similar to those used earlier in their synthesis of ODNs by the phosphotriester method. In other words, the support-bound material was separated from the low molecular weight substances by precipitation from Et_2_O and recrystallization from a mixture of MeCN and Et_2_O. In this case, four precipitation/recrystallization steps were needed in each coupling cycle: after detritylation, coupling, capping and oxidation (Scheme 4). The building blocks were base-moiety protected 5´-*O*-DMTr-nucleoside 3´-(2-cyanoethyl-*N*,*N*-diisopropylphosphoramidites), i.e., the ones used in standard solid-supported synthesis. Phosphite triesters were oxidized to phosphate triesters after each coupling with *tert*-butyl hydroperoxide in MeCN [52]. On using 2.5 equiv of the phosphoramidite block and 10 equiv of tetrazole as an activator in MeCN, 98–99% coupling yields were obtained. Support-bound octamer, DMTr-d(5´-TAGCGCTA-3´)-PEG could be obtained in 93% yield and a 20-mer in 85% yield. These yields are surprisingly high, requiring 99% yield per coupling cycle. Release/deprotection by conventional ammonolysis followed by acidolytic detritylation and removal of the PEG-support by precipitation was reported to give the pure octamer in 50% higher yield than the phosphotriester approach.

The essentially same approach was later applied to the synthesis of a PEG-conjugated 12-mer antisense ODN [53] and a 13-mer purine-rich triple-helix forming sequence [54]. Immobilization of the 3´-terminal nucleosides via a succinyl linker was, however, replaced by direct phosphoramidite coupling to the terminal OH of PEG, which gave a stable phosphodiester linkage upon ammonolytical deprotection. In other words, the ODNs were used as PEG-conjugates in biological studies. In addition, a bifunctionalized PEG, bearing the acid labile DMTrO group at one end and a base labile Fmoc-NH functionality at the other end, has been used as a soluble support to obtain oligonucleotide–PEG–peptide conjugates [55–56]. The Fmoc protecting group was first removed and the peptide was assembled on the exposed amino function. Since the peptide moiety did not contain acid labile side chain protections, the oligonucleotide sequence could then be assembled by the protocol discussed above.

**Scheme 5:**
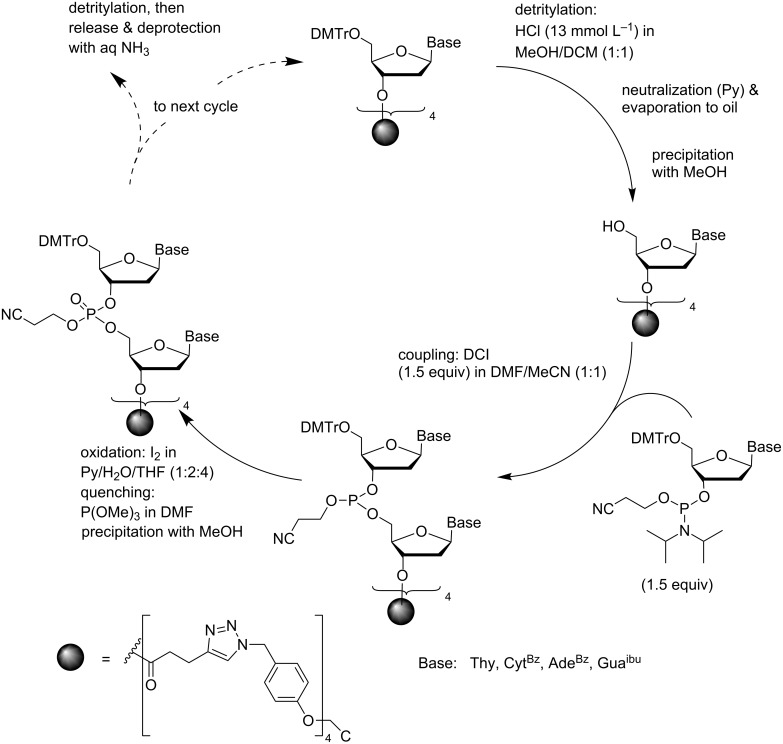
Synthesis of ODNs on a precipitative tetrapodal support by conventional phosphoramidite chemistry [43].

Another precipitative support that has been used for the synthesis of ODNs is the tetrapodal tetrakis-*O*-[4-(azidomethyl)phenyl)]pentaerythritol-derived support discussed above [43]. Two precipitations from MeOH were carried out in each coupling cycle: one after the 5´-O-detritylation and the second after the coupling/oxidation step (Scheme 5). The detritylation was carried out with HCl in a 1:1 (v/v) mixture of MeOH and DCM under carefully controlled conditions. The acid was neutralized with slight excess of pyridine. To prevent re-tritylation of the exposed 5´-OH by trityl carbocation, prolonged heating of the oily residue was avoided. Precipitation from MeOH quantitatively removed the traces of the DMTr carbocation as a methyl ether. Couplings were carried out in a 1:1 (v/v) mixture of DMF and MeCN using standard phosphoramidite building blocks (1.5 equiv) and 4,5-dicyanoimidazole (DCI, 1.5 equiv) as an activator. The resulting phosphite triesters were converted to phosphate esters by conventional aq iodine oxidation. Precipitation by dilution with MeOH removed all traces of reagents and monomeric nucleoside derivatives. As a proof of concept, a pentamer, d(5´-AGCCT-3´), was assembled. Release and deprotection of the oligomer by conventional ammonolysis were accompanied by precipitation of the support. The pentamer, homogeneous by HPLC, was obtained in a 43% yield.

**Scheme 6:**
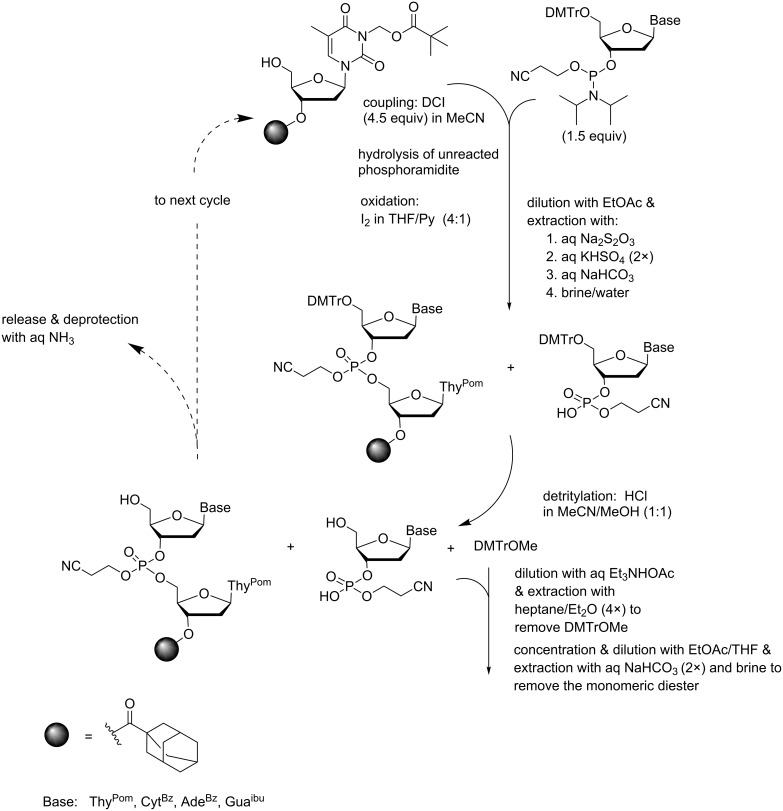
Synthesis of ODNs by an extractive strategy on an adamant-1-ylacetyl support [57].

Besides precipitation, extraction offers a possible approach for the separation of the soluble-supported oligonucleotides from small molecular materials. The underlying idea is to keep the growing oligonucleotide chain sufficiently hydrophobic to enable removal of the excess of building blocks, activators and wastes by water extraction, but still allow removal of highly hydrophobic substances, above all DMTrOMe, by extraction with very nonpolar solvents. The feasibility of this concept has been demonstrated by assembling a hexamer, d(5´-ATGCTT-3´), on 3´-(*O*-adamant-1-yl)acetyl-3-pivaloyloxymethylthymidine [57]. Twelve individual extractions had to be carried out in each synthetic cycle, as indicated in Scheme 6. First, DCI activated coupling in MeCN, hydrolysis of the unreacted phosphoramidite and subsequent I_2_ oxidation in aq THF/pyridine was followed by dilution with EtOAc and washing with aq Na_2_S_2_O_3_, aq KHSO_4_ (twice), aq NaHCO_3_ and brine. After HCl catalyzed detritylation in a 6:1 mixture of MeOH and MeCN, another set of extraction was performed. The mixture was neutralized with Et_3_NHOAc and diluted with aq MeCN to give a 2:2:1 mixture of MeCN, MeOH and H_2_O. The DMTrOMe byproduct was first removed by extracting four times with a 2:1 mixture of heptane and Et_2_O. The polar phase was concentrated, diluted with a 5:2 mixture of EtOAc and THF, and washed twice with aq NaHCO_3_ and then with diluted brine. Standard base moiety protections (dA^Bz^, dC^Bz^, dG^ibu^) were employed, with the exception of thymine, which was used as a 3-pivaloyloxymethyl derivative to ensure sufficient hydrophobicity. On using 1.5 equiv of the phosphoramidite for coupling, the fully protected hexamer was obtained in 67% yield. Ammonolysis and ion-exchange chromatographic purification then gave hexamer d(5´-ATGCTT-3´) in isolated 39% yield.

**Scheme 7:**
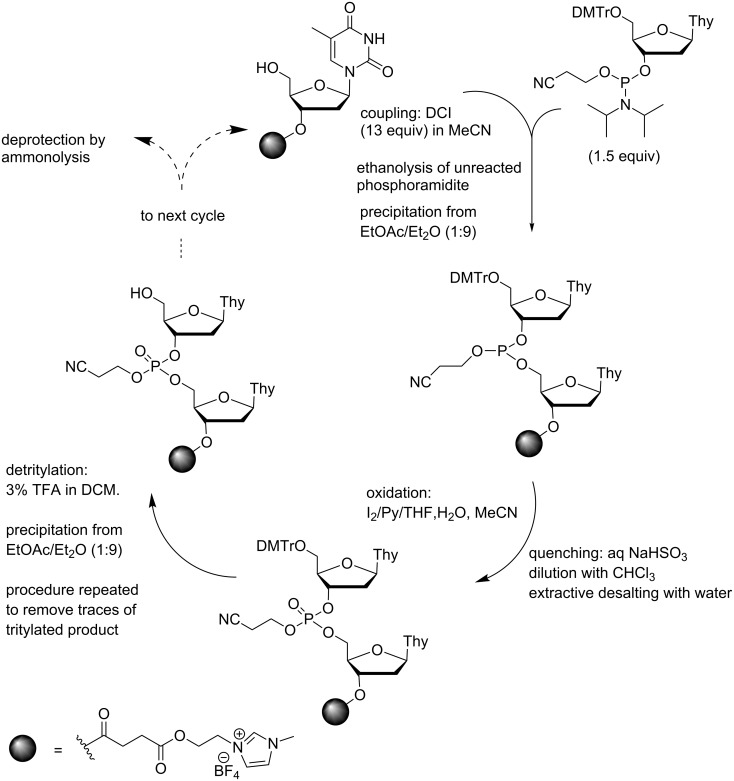
Synthesis of ODNs by a combination of extractive and precipitative strategy [58].

Esterification of a 5´-*O*-DMTr-3´-*O*-succinylthymidine with 3-(2-hydroxyethyl)-1-methyl-1*H*-imidazol-3-ium tetrafluoroborate has given another soluble support that allows utilization of extractive techniques, in this case in combination with precipitation [58] (Scheme 7). The support precipitates from a 1:9 mixture of EtOAc and Et_2_O, but is soluble in chloroform, which allows removal of salts by extraction with water. The couplings were carried out with 1.5 equiv of standard 2-cyanoethyl-*N*,*N*-diisopropylphosphoramidites in THF or MeCN, using DCI as an activator. Unreacted phosphoramidites were quenched by EtOH and the support was precipitated before the oxidation step, repeatedly when needed. The precipitate was dissolved in MeCN and conventional aq I_2_ oxidation was performed. After bisulfite quenching, the mixture was diluted with chloroform and washed with water to remove salts. The organic phase was evaporated to foam and subjected to detritylation with TFA in DCM or MeCN. The detritylated material was then precipitated with the EtOAc/Et_2_O mixture. The product was, however, still partly tritylated, and the detritylation had therefore to be repeated. The longest oligomer synthesized was a thymidine tetramer. The yield of the support-bound tetramer was 87%, but no isolated yield was reported.

**Scheme 8:**
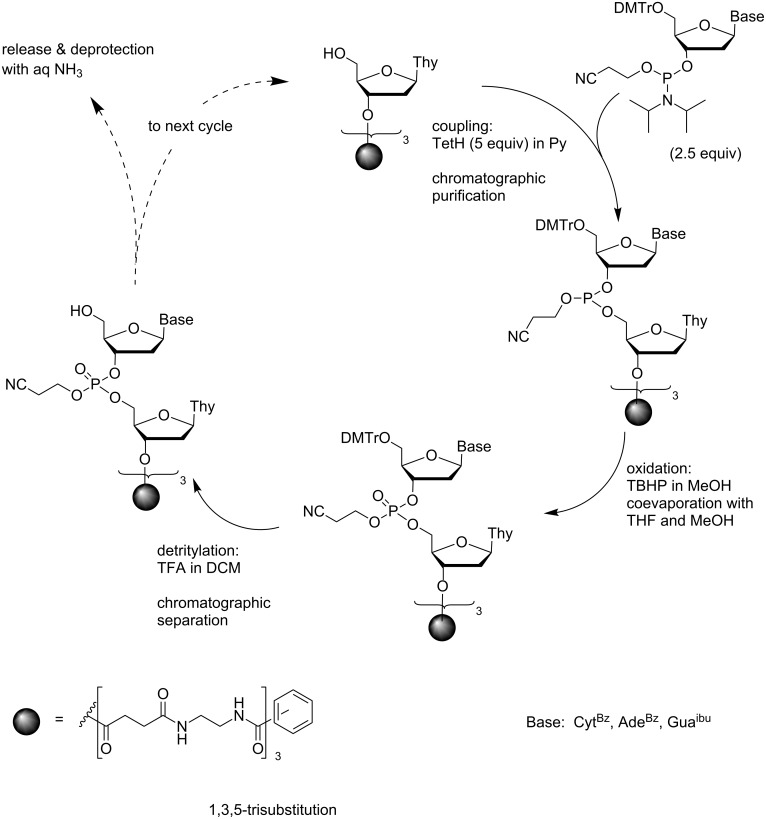
Synthesis of ODNs by phosphoramidite chemistry on a *N*^1^,*N*^3^,*N*^5^-tris(2-aminoethyl)benzene-1,3,5-tricarboxamide support by making use of chromatographic separation [59].

Although chromatographic separation appears to be a tedious procedure compared to precipitation or extraction, it has been successfully applied to the synthesis of ODNs on a soluble support. The studies of Wörl and Köster on *N*^1^,*N*^3^,*N*^5^-tris(2-aminoethyl)benzene-1,3,5-tricarboxamide derivatized with 3´-*O*-succinylthymidine offered an early example [59] (Scheme 8). Owing to poor solubility of the support into MeCN, elongation of the branches by tetrazole promoted coupling of nucleoside phosphoramidites was carried out in pyridine under argon. On using 2.5 equiv of the phosphoramidite and 5 equiv of tetrazole, the average coupling yield was 96%. The mixture was concentrated and subjected to gel permeation chromatography in MeOH to remove the low molecular weight compounds. The pooled fractions containing the support-bound oligonucleotides were concentrated and oxidized with *tert*-butyl hydroperoxide. The excess of oxidizing agent was removed by coevaporation with THF and MeOH, and the residue was dissolved into an 80:19:1 mixture of DCM, MeNO_2_ and MeOH. Finally, the 5´-terminal DMTr groups were removed by adding 2% TFA. After neutralization with Et_3_N, the chromatographic separation was repeated. Upon assembly of a fully protected 10-mer, d(5´-*O*-DMTr-G^ibu^A^Bz^C^Bz^G^ibu^G^ibu^C^Bz^C^Bz^A^Bz^G^ibu^T)_3_-support, the average yield of an entire coupling cycle was 87% and the overall yield 33%. Conventional ammonolysis was used for the release from the support. Since no capping reaction had been carried out in any coupling cycle, the n − 1 fragment was formed in a considerable amount. Assembly from dimeric phosphoramidites was additionally attempted, but the chromatographic separation was not efficient enough to remove the excess of the dimeric building block.

Much later chromatographic separation was exploited for the assembly of short ODNs from base-moiety-protected 5´-(1-methoxy-1-methylethyl)-2´-deoxyribonucleoside 3´-phosphoramidites on a fully methylated β-cyclodextrin support [60]. The 1-methoxy-1-methylethyl group may be removed by acid-catalyzed methanolysis approximately as readily as the DMTr group, but it gives only volatile products. Accordingly, after removal of the 5´-protection, only evaporation was needed. The subsequent flash chromatographic purification was, in turn, rather straightforward owing to the hydrophobic support. After ammonolytic release and deprotection, the methylated cyclodextrin support could be removed by simple extraction with DCM. A pentameric oligonucleotide, 5´-TACTT-3´, was obtained in 52% yield on using 1.5 equiv of phosphoramidites and 1.5 equiv of DCI as an activator.

### Synthesis of oligoribonucleotides by the phosphoramidite chemistry

**Scheme 9:**
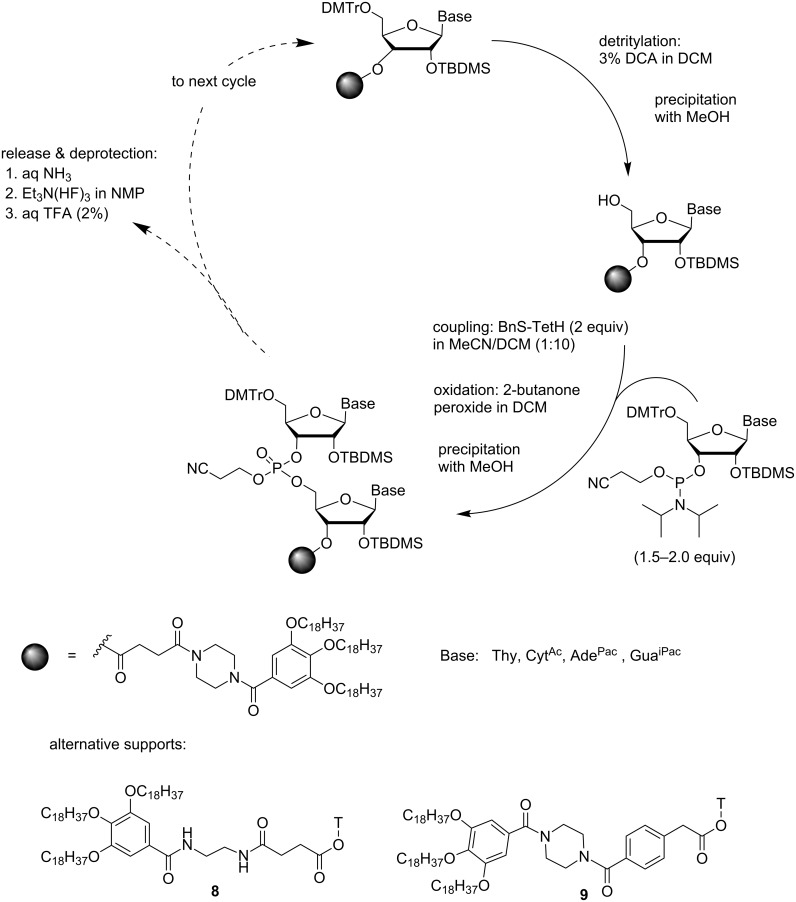
Synthesis of ORNs by phosphoramidite chemistry on a hydrophobic support [61].

Three different protocols, all based on separation of the support-bound oligonucleotide from low-molecular weight compounds by precipitation, have been utilized for the synthesis of oligoribonucleotides by phosphoramidite chemistry. A highly hydrophobic support that is well soluble in THF, CHCl_3_ and DCM, but insoluble in MeOH, MeCN and EtCN, has been used to assemble a 21-mer RNA sequence in gram scale [61] (Scheme 9). First, the DMTr group was removed with DCA in DCM and the detritylated support was precipitated from MeOH. A base-moiety-protected (A^Pac^, G^iPac^, C^Ac^) 5´-*O*-DMTr-2´-*O*-TBDMS-nucleoside 3´-(2-cyanoethyl-*N*,*N*-diisopropylphosphoramidite) (1.5–2.0 equiv) was then coupled in a 1:10 mixture of MeCN and DCM using 5-(benzylthio)-1*H*-tetrazole as an activator. After completion of the coupling, oxidation to the phosphate ester was carried out in the same pot by addition of 2-butanone peroxide in DCM. Dilution with MeOH precipitated the support. With 15–21-mer oligomers, some support-bound material, however, remained in solution and was recovered by adsorption to C18-coated silica gel. The cycle was completed by detritylation with DCA (3%) in DCM. Cleavage and deprotection was conventional: ammonolysis in aqueous EtOH, followed by desilylation with Et_3_N(HF)_3_ in *N*-methylpyridinone (NMP) and removal of the 5´-*O*-DMTr with aq TFA (2%). The isolated yield, 26%, is surprisingly high, taking into account that the synthesis involves more than 60 steps. In fact, the fully protected sequence was reported to be obtained in 46% yield, which corresponds to 98% yield per coupling cycle. Evidently the lack of amide hydrogens on the support is essential for the desired solubility properties, since replacement of the piperazine fragment within the linker structure with ethylene diamine **8** gave considerably less satisfactory results.

When the succinyl linker was replaced with the 4-carboxymethylbenzoic acid linker **9**, the fully protected oligomer could be released by catalytic hydrogenation. This allowed the preparation of appropriately protected dimeric and trimeric building blocks having only the 3´-terminal hydroxy function unprotected and, hence, subject to phosphitylation [62].

**Scheme 10:**
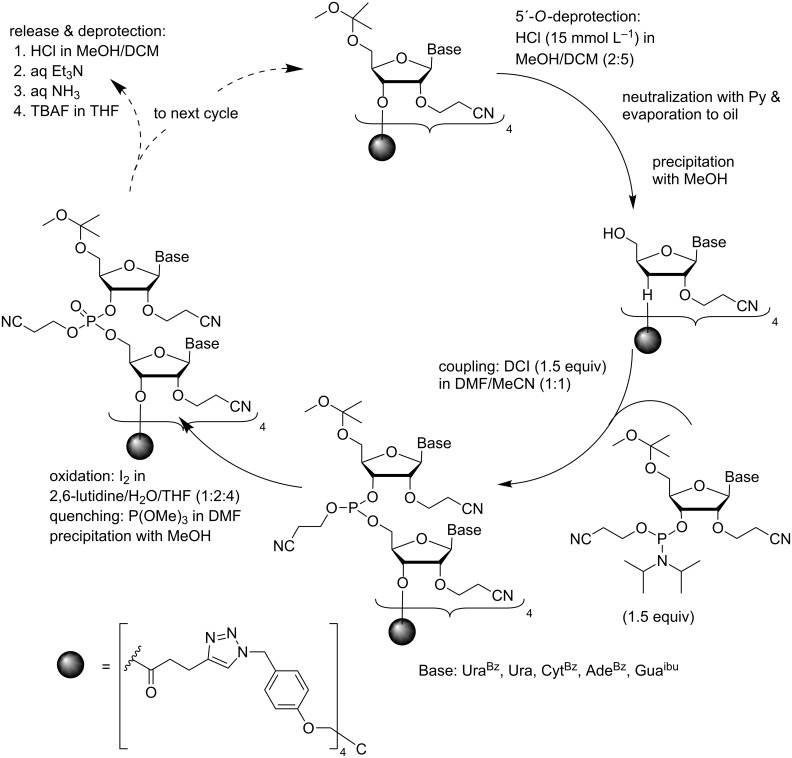
Synthesis of ORNs by the phosphoramidite chemistry on a precipitative tetrapodal support using 2´-*O*-(2-cyanoethyl)-5´-*O*-(1-methoxy-1-methylethyl) protected building blocks [63].

The terapodal tetrakis-*O*-[4-(azidomethyl)phenyl]pentaerythritol-derived support has also been used for the synthesis of short ORNs [63] (Scheme 10). Unusual 2´-*O*-(2-cyanoethyl)-5´-*O*-(1-methoxy-1-methylethyl)ribonucleoside 3´-phosphoramidites were used, since common commercially available building blocks turned out to be too hydrophobic to allow precipitation of the support-bound oligonucleotides from MeOH. The 1-methoxy-1-methylethyl group could be removed quantitatively as a dimethyl acetal of acetone upon acid-catalyzed transesterification in MeOH. The 3'-terminal nucleoside was attached to the support as a 3´-*O*-(4-pentynoyl) derivative, essentially as with 2´-deoxyribonucleosides. The acid-catalyzed removal of the 5´-*O*-1-methoxy-1-methylethyl group by 0.015 mol L^−1^ HCl in MeOH was essentially as fast as that of the DMTr group and no additional scavengers were needed to push the reaction to completion. Precipitation of the support from cold MeOH was quantitative. The phosphoramidite blocks were used in 50% excess and the coupling was promoted with DCI in a mixture of MeCN and DMF (1:1, v/v) under N_2_. The phosphite triester obtained was oxidized to phosphate triester by conventional aqueous iodine treatment. The support was separated from all small molecular reagents by concentration to oil and subsequent precipitation from cold MeOH. Finally, the support-bound ORNs were subjected to consecutive treatments with triethylamine, ammonia and with TBAF. The fully deprotected ORNs were precipitated with NaOAc from EtOH. The hexamer, 5'-ACGUUU-3', was obtained in 54% yield, which means that the average coupling yield was 86%.

**Scheme 11:**
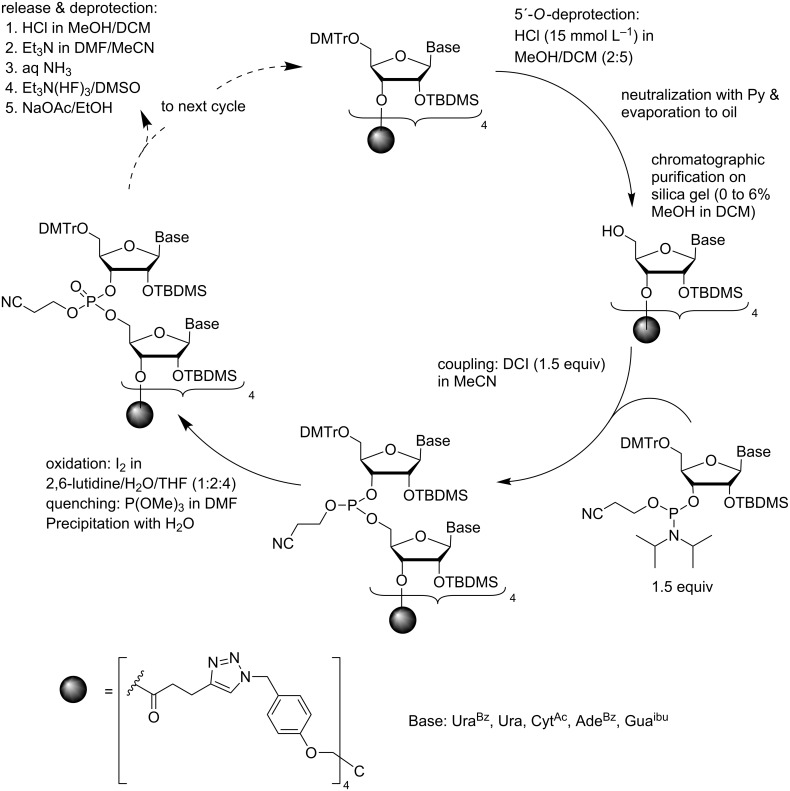
Synthesis of ORNs by phosphoramidite chemistry on a precipitative tetrapodal support from commercially available building blocks [64].

When 5´-*O*-DMTr-2´-*O*-TBDMS protected building blocks were used [64], instead of two precipitations from MeOH, each coupling cycle involved one precipitation from water and one flash chromatography (Scheme 11). Detritylation was carried out with HCl in a 2:5 (v/v) mixture of MeOH and DCM. The acid was neutralized with pyridine, the mixture concentrated to oil and subjected to flash column chromatography on silica gel. For subsequent coupling, the desired commercial block was used in 50% excess and DCI as an activator. After standard I_2_ oxidation, the support-bound material was precipitated from water. The precipitation was quantitative, but some reagents and byproducts, above all DCI, coprecipitated with the support. The flash chromatography after next detritylation, however, removed these impurities. It is worth noting that the hydrophobic support greatly facilitated the chromatographic separation. After completion of the chain assembly, treatment with Et_3_N, followed by ammonolysis and finally Et_3_N(HF)_3_ treatment, released the ORN, which was precipitated from cold MeOH with NaOAc. By this method, pentamer 5´-AGCUU-3´ was prepared in 46% yield.

### Synthesis of oligodeoxyribonucleotides by the alkyl *H*-phosphonate chemistry

**Scheme 12:**
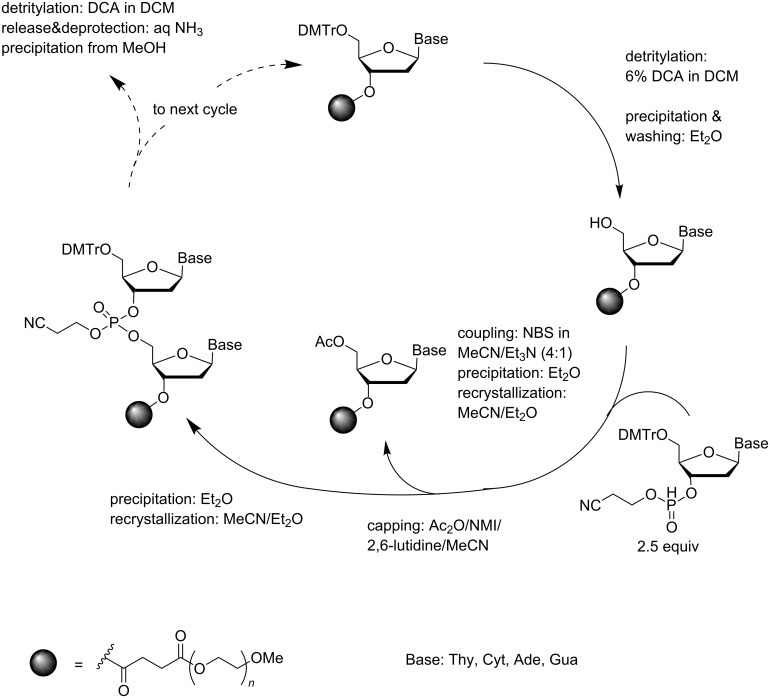
Synthesis of ODNs on a precipitative PEG-support by *H*-phosphonate chemistry [65].

Surprisingly few attempts have been made to apply the *H*-phosphonate chemistry to the soluble-supported synthesis of oligonucleotides and most of these attempts have concerned the preparation of phosphorothioate ODNs, as discussed below. The only successful synthesis of unmodified ODNs was based on oxidative coupling of alkyl *H*-phosphonates on a PEG support [65]. The 3´-terminal nucleoside was immobilized to a PEG support via a succinyl linker, detritylated with DCA in DCM and precipitated and washed with Et_2_O (Scheme 12). 3´-(2-Cyanoethyl *H*-phosphonate)s of 5´-*O*-DMTr-2´-deoxyribonucleosides were then used as synthons for the chain elongation. The oxidative couplings were carried out in a 4:1 (v/v) mixture of MeCN and Et_3_N using *N*-bromosuccinimide (NBS) as an activator. The coupling efficiency was high (98%) on using 2.5 equiv of the *H*-phosphonate synthon and 5 equiv of the activator. After each coupling step, the support was precipitated from Et_2_O and recrystallized from MeCN/Et_2_O. The unreacted hydroxy groups were capped by acetylation and the support was again precipitated with Et_2_O. Finally, ammonolysis was carried out and the oligonucleotide was separated from the PEG support by precipitation from MeOH. The feasibility of the method was tested by the synthesis of d(5´-ACGGGCCCGT-3´) in 75% yield.

### Synthesis of oligonucleotide phosphorothioates

Phosphorothioate oligonucleotides have largely been synthesized by the same approaches as their oxygen counterparts. In fact, the only major difference is that oxidative sulfurization has been applied instead of oxidation. For example, when the phosphoramidite chemistry on a precipitative PEG support was applied, tetraethylthiuram disulfide (TETD; 0.5 mol L^−1^ in MeCN; 10-fold excess) was used as the sulfurization reagent [66] instead of *tert*-butyl hydroperoxide used for the oxidation in the synthesis of unmodified ODNs [51]. On using 2.5 equiv of phosphoramidites for coupling, a support-bound 20-mer was obtained in 83% yield, and the pure oligomer could be isolated from the crude in 55% yield.

As a modification of this approach, phosphorothioate ODNs have been prepared by using 3´-phosphoramidites of dinucleoside-3´,5´-phosphorothioates as building blocks. The coupling efficiency was 99% on using 3.0 equiv of the dimeric building block [67]. The resulting phosphite triester was after each coupling oxidatively sulfurized with a 10-fold excess of diethyldithiocarbonate disulfide (DDD) [68]. The capping step after each sulfurization was carried out at 0 °C to avoid cleavage of the 2-cyanoethyl groups from the phosphorothioate triester linkages. Methyl *tert*-butyl ether was used for precipitations after detritylation and coupling/sulfurization steps. Detritylation with DCA in DCE, however, turned out to be somewhat problematic, since the procedure had often to be repeated. Conventional ammonolysis was used for the release from support and removal of base and phosphate protections. By this approach, a 15-mer phosphorothioate ODN (sequence not given, one G coupled as a monomer) was synthesized in 58% overall yield.

**Scheme 13:**
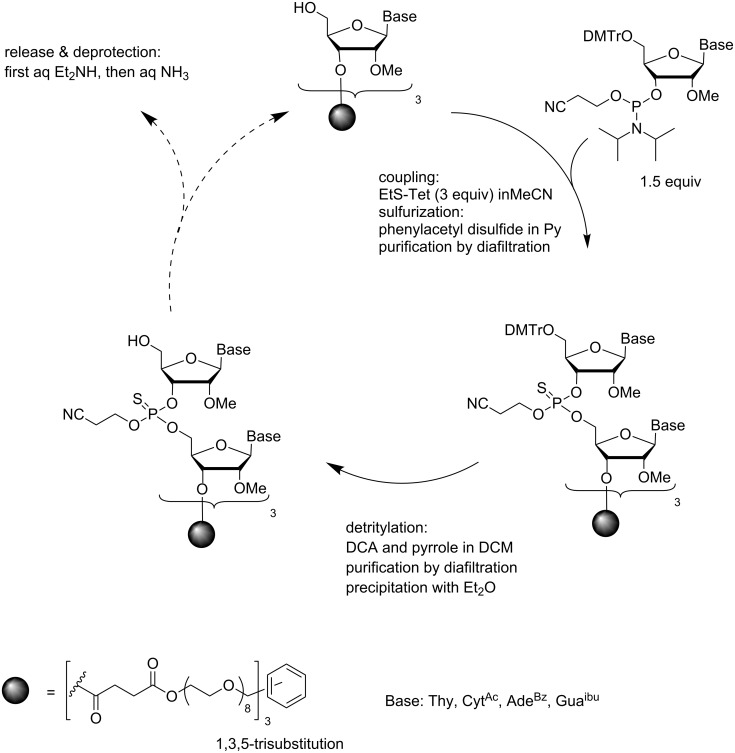
Synthesis of 2´-*O*-methyl ORN phosphorothioates by phosphoramidite chemistry by making use of nanofiltration in organic solvents [69–70].

The development of new materials that allow nanofiltration in organic solvents has offered an entirely new paradigm for the soluble-supported synthesis of oligonucleotides. The underlying idea is that on passing the reaction mixture by high pressure through a membrane, small molecules pass through the membrane, while the support is too bulky to escape through the nanopores of the membrane material. As a proof of concept, a 9-mer 2´-*O*-methyl oligoribonucleotide phosphorothioate has been synthesized [69–70]. The soluble support was 1,3,5-tris(hydroxymethyl)benzene derivatized with an eight units long PEG chain (Scheme 13), called homostar by the authors. The 3´-terminal nucleoside, in this case 5´-*O*-DMTr-2´-*O*-methyluridine, was attached via a succinyl linker to the terminal hydroxy functions of the support. Commercially available 5´-*O*-DMTr-2´-*O*-methylribonucleoside 3´-(2-cyanoethyl-*N*,*N*-diisopropylphosphoramidites (U, C^Ac^, G^ibu^, A^Bz^) were employed for chain elongation. Ethylthiotetrazole-activated coupling (3 equiv per OH) in MeCN was followed by sulfurization with phenylacetyl disulfide in pyridine. All small molecule compounds were removed by the so-called diafiltration through a polybenzimidazole-based membrane PBI-17DBX [71–72]. In other words, the volume of the reaction mixture was kept unchanged during the filtration by continuous addition of pure solvent. After changing the solvent to DCM, detritylation with dichloroacetic acid was performed using pyrrole as a scavenger for the DMTr cation [73]. It turned out, however, that the DMTr-pyrrole formed could not be entirely removed by filtration, but a precipitation of the support with Et_2_O was required for quantitative removal of this impurity. During the first four coupling cycles, the coupling yields gradually increased from 75 to 90%, and remained after that high (90–95%). Isolation of pure deprotected 9-mer, however, required HPLC purification and could be obtained in only 16% yield calculated from the crude support-bound material.

## Conclusion

Several approaches based on precipitation, extraction, chromatographic separation or nanofiltration of a soluble support have been developed by making use of phosphoramidite, phosphotriester or *H*-phosphonate coupling. Usually these methods are aimed to be utilized for an industrial-scale synthesis of oligonucleotides. However, none of them has really tested at such a scale that the feasibility of industrial use could be critically judged. The method based on nanofiltration in organic solvents clearly differs from the other approaches and represents a genuine effort towards an industrial process. The results still are very preliminary and the success will undoubtedly depend on further development of the membrane material and efficiency of the recyclization of the large solvent amounts.

All the other approaches discussed above allow lab-scale syntheses of oligonucleotides used for research purposes in gram scale. The advantage of the proposed soluble support strategies is that no special equipment is needed, and hence, they evidently are of interest for research groups that only now and then require large amounts of oligonucleotides for research in their main field. Comparison of the applicability of these methods is difficult on the basis of the data available. One interesting point is that some of the methods use capping, as usually on a solid support, whereas others omit it. None of the groups has carried out comparative studies that would shed light on the necessity of capping. Capping increases the number of manipulation but evidently simplifies the final purification. Which one is more important? Similarly, the phosphoramidite coupling is more efficient than phosphortriester or oxidative *H*-phosphonate coupling, but requires a separate oxidation step. Which one is more important, high coupling efficiency or simpler coupling cycle? Finally, it is worth noting that all the strategies proposed so far are based on acid-labile 5´-*O*-protection, although it inevitably leads to depurination as a side reaction, in particular on using acyl protections for the amino functions. May be a proper solution to this old problem would open doors for success of oligonucleotide synthesis on a soluble support.

## Abbreviations

**Table 1:** List of abbreviations.

A	adenosine
Ade	adenine
BnS-TetH	5-benzylthiotetrazole
Cyt	cytosine
dA	2´-deoxyadenosine
dC	2´-deoxycytidine
DCE	1,2-dichloroethane
DCI	4,5-dicyanoimidazole
DCM	dichloromethane
DDD	diethyldithiocarbonate disulfide
DEAE	2-(diethylamino)ethyl
dG	*2´-deoxyguanosine*
DMF	*N*,*N*-dimethylformamide
DMTr	4,4´-dimethoxytrityl
Dpa	diphenylacetyl
dT	thymidine
EtS-TetH	5-ethylthiotetrazole
G	guanosine
Gua	guanine
ibu	isobutyl
iPac	4-isopropylphenoxyacetyl
MMTr	4-methoxytrityl
MSNT	1-(mesitylene-2-sulfonyl)-3-nitro-1,2,4-triazole
NBS	*N*-bromosuccinimide
NMI	*N*-methylimidazole
NMP	*N*-methyl-2-pyrrolidone
ODN	oligodeoxyribonucleotide
ON	oligonucleotide
ORN	oligoribonucleotide
Pac	phenoxyacetyl
PEG	polyethylene glycol
PG	protecting group
Pom	pivaloyloxymethyl
Py	pyridine
TBAF	tetrabutylammonium fluoride
TBDMS	*tert*-butyldimethylsilyl
TBHP	*tert*-butyl hydroperoxide
TCA	trichloroacetic acid
TEA	triethylammonium
TetH	tetrazole
TETD	tetraethylthiuram disulfide
THF	tetrahydrofuran
Thy	thymine
TOM	triisopropylsilyloxymethyl
U	uridine
Ura	uracil
